# Antileishmanial and Immunomodulatory Activity of Paclitaxel and Docetaxel Combined with Miltefosine and Paromomycin

**DOI:** 10.3390/ijms27073055

**Published:** 2026-03-27

**Authors:** Estela Melcón-Fernández, Rafael Balaña-Fouce, Carlos García-Estrada, Rosa M. Reguera, Celia Fernández-Rubio, Marta Cendón-Álvarez, Yolanda Pérez-Pertejo

**Affiliations:** 1 Departamento de Ciencias Biomédicas, Facultad de Veterinaria, Universidad de León, 24071 León, Spain; emelf@unileon.es (E.M.-F.); rbalf@unileon.es (R.B.-F.); cgare@unileon.es (C.G.-E.); rmregt@unileon.es (R.M.R.); cferrb@unileon.es (C.F.-R.); mcenda00@estudiantes.unileon.es (M.C.-Á.); 2 Instituto de Biomedicina (IBIOMED), Universidad de León, Campus de Vegazana s/n, 24071 León, Spain

**Keywords:** leishmaniasis, drug combinations, drug repurposing, paclitaxel, docetaxel, miltefosine, paromomycin, cytokines

## Abstract

A wide range of Leishmania species, transmitted by phlebotomine, cause leishmaniasis, which presents diverse clinical manifestations. Leishmaniasis has a high impact on vulnerable communities, primarily affecting people suffering from malnutrition or poor housing. Because leishmaniasis is associated with poverty, access to treatment is limited. In addition, high drug toxicity and therapeutic failure, related to drug resistance, remain major challenges. Therefore, there is a need to develop new therapeutic approaches that are safer and more effective. Drug combinations and repurposing are two strategies used in the development of treatments. The combination of drugs with different mechanisms of action can minimise resistance and allow dose reduction, increasing the likelihood of successful drug repurposing. This study evaluated the antileishmanial effects of combining antitumoral agents (paclitaxel and docetaxel) with standard drugs (miltefosine and paromomycin). Results demonstrated synergistic effects at higher doses. Furthermore, the antitumoral compounds enhanced the host immune response by promoting macrophage polarisation toward the M1 phenotype, essential for parasite control. These findings highlight a promising approach that could improve efficacy and reduce resistance.

## 1. Introduction

According to the 2024 Drugs for Neglected Diseases initiative (DNDi) annual report (dndi.org), more than one billion people worldwide are at risk of leishmaniasis, with children representing nearly half of all reported cases. Leishmaniasis is a neglected tropical disease caused by approximately 20 species of the genus *Leishmania*, and its clinical manifestations vary depending on the parasite species and the host immune response [1]. Transmission occurs through the bite of infected female phlebotomine sand flies, making the disease highly sensitive to environmental and climatic factors. In this context, climate change is contributing to the expansion of vector populations into previously non-endemic regions, including northern Europe [2]. Socioeconomic determinants such as malnutrition, poor housing conditions, and population displacement also shape the epidemiology of leishmaniasis, disproportionately affecting vulnerable populations (dndi.org). Consequently, the European Centre for Disease Prevention and Control (ECDC) has developed contingency plans, aimed at preventing the spread of the disease within the European Union [3].

Leishmaniasis presents several clinical forms. Visceral leishmaniasis (VL), also known as kala-azar, is the most severe, characterised by hepatosplenomegaly, fever, and weight loss, and is fatal if left untreated, with an estimated 50,000–90,000 new cases annually worldwide. Cutaneous leishmaniasis (CL) is the most prevalent form, causing ulcerative skin lesions that may result in permanent scarring, leading to physical disability and social stigma, with 600,000–1,000,000 new cases each year. Mucocutaneous leishmaniasis (MCL) produces the partial or total destruction of mucous membranes of the nose, mouth, and throat. Post-kala-azar dermal leishmaniasis (PKDL) is a dermatological sequela of VL that can develop months to years after successful VL treatment; although non-lethal, PKDL is disfiguring and sustains transmission, contributing to social stigmatisation [4].

Despite the significant global burden of these diseases, therapeutic options remain limited. Sodium stibogluconate (SSG) and amphotericin B, administered as the deoxycholate salt or as a liposomal formulation AmBisome^®^ (L-AMB), are the drugs of choice for VL. These therapies require parenteral administration and often necessitate hospitalisation. Moreover, amphotericin B formulations, particularly the liposomal derivative (AmBisome^®^; L-AMB), are associated with high treatment costs, which significantly limit their widespread use, especially in low-and middle-income countries [5]. Miltefosine (MTF), approved in 2004, is the only orally available antileishmanial drug. However, its use is associated with teratogenic effects, which preclude its administration during pregnancy. Paromomycin (PMM), approved in 2006, shows limited efficacy as monotherapy and is therefore mainly used in combination with SSG or with MTF [6].

The emergence of resistance further hinders disease eradication. In India, the prolonged use of SSG has led to the selection of resistant parasite strains, significantly reducing its clinical utility [7,8]. Similarly, resistance to MTF has also been increasingly reported [9] and multiple resistance events have been documented in the Mediterranean region, underscoring the global dimension of this challenge [10]. For these reasons, the discovery of novel antileishmanial drugs and the optimisation of treatment regimens are necessary and urgent [11]. Among the approaches promoted by the DNDi, the development of shorter and safer treatments based on combinations of existing drugs has been prioritised. Combination therapies can enhance therapeutic efficacy, reduce toxicity and limit the emergence of drug resistance [12,13]. In addition, they increase the potential of drug repurposing strategies, as effective treatments may be achieved at lower plasma concentrations. The use of repurposed drugs also benefits from the prior knowledge of safety profiles and pharmacokinetic properties, which can substantially reduce both the cost and time required for drug development and regulatory approval [14,15,16,17].

Among the potential candidates identified through repurposing strategies, microtubule-targeting agents have attracted increasing attention. Taxanes are microtubule-stabilising agents that bind to β-tubulin and promote the polymerisation and stabilisation of cellular microtubules, thereby disrupting microtubule dynamics and interfering with cell division. Among them, paclitaxel (PTX) and docetaxel (DTX) are widely used antitumoral drugs that also demonstrate antileishmanial activity in repurposing studies based on large drug collections [17]. The effects of tubulin inhibitors on the parasite cell cycle have been reported in several studies, supporting tubulin as a relevant molecular target in *Leishmania* [18,19]. In addition, alterations in tubulin expression in resistant parasites increase their sensitivity to tubulin-targeting compounds [20,21]. Beyond their direct antiparasitic activity, taxanes also exert immunomodulatory effects. The activation of Th1 responses and the polarisation of macrophages toward the M1 phenotype are essential for parasite control [22,23,24,25,26]. In this context, PTX activates infected macrophages and promotes parasite killing through nitric oxide (NO) production, while PTX, DTX and related agents induce the expression of pro-inflammatory genes [27,28]. Given their dual antiparasitic and immunomodulatory properties, taxanes represent attractive candidates for combination therapies aimed at enhancing efficacy while reducing drug doses and toxicity.

Based on these findings, and in the search for improved antileishmanial therapies, we evaluated the antiparasitic effects of combinations of MTF and PMM with PTX and DTX using both in vitro (axenic amastigotes) and ex vivo (intramacrophagic amastigotes) models. Additionally, we assessed the potential immunomodulatory effects of these drug combinations.

## 2. Results

The antiparasitic activity of the taxanes paclitaxel (PTX) and docetaxel (DTX) was evaluated both as single agents and in combination with miltefosine (MTF) or paromomycin (PMM) against iRFP *L. infantum*. Experiments were conducted using intramacrophagic amastigotes isolated from the spleen and axenic amastigotes recovered from bone marrow cells of infected Balb/c mice, as described in the Section 4. The infrared fluorescence emitted by the cultures exposed to drugs or drug combinations was used to calculate parasite viability following exposure. Both amastigote models were included to account for the potential influence of macrophage activation on taxane activity, as previous studies have reported significant differences between these experimental platforms [29].

### 2.1. Leishmanicidal Effect of PTX and DTX in Combination with MTF

**Figure 1:**
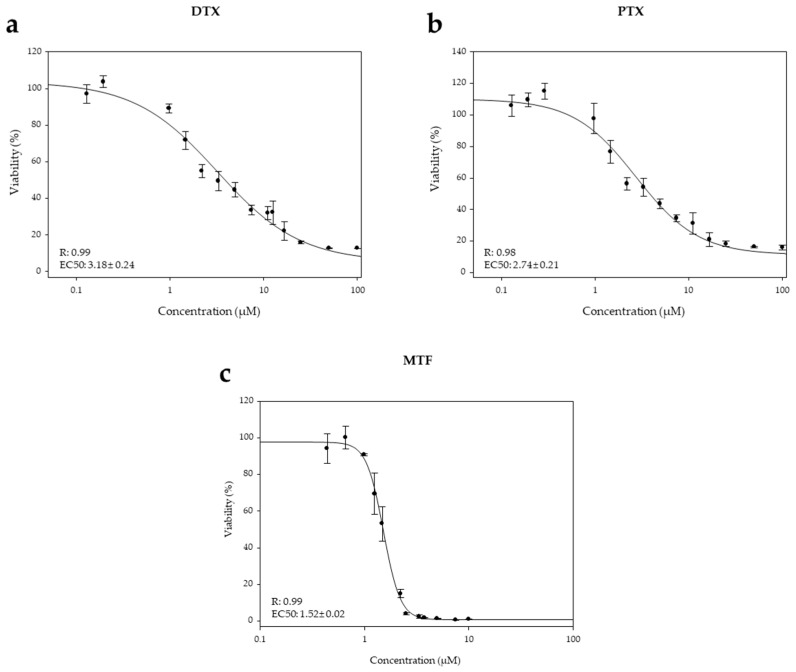
Dose–response curves of DTX (**a**), PTX (**b**) and MTF (**c**) against iRFP-*L. infantum* amastigotes obtained from the bone marrow cells of infected Balb/c mice. Serial dilutions of DTX and PTX (^1^/_2_ and ^2^/_3_ dilutions) were prepared, from a starting concentration of 100 µM. MTF was serially diluted (^2^/_3_ dilutions) from 10 µM. Dose–response curves were fitted using SigmaPlot 10.1 statistical package. Results represent the mean ± SD of three independent experiments with five technical replicates each.

First, the EC_50_ values for taxanes and MTF, when applied individually to axenic amastigotes for 72 h were determined (Figure 1). PTX and DTX exhibited similar potencies, with EC_50_ values of 2.74 ± 0.21 µM and 3.18 ± 0.24 µM respectively, whereas the EC_50_ for MTF was 1.52 ± 0.02 µM.

Given the similarity of the EC_50_ values, the combination ratios selected to assess the drug combinations for MTF/PTX and MTF/DTX in bone marrow axenic amastigotes were 1:1, 1:2 and 2:1. The interactions between drugs in these combinations were studied using the Chou–Talalay method implemented in the ‘CalcuSyn’ software. Thus, the combination index (CI), was calculated with CI = 1 indicating additive effect, CI < 1 indicating synergism and CI > 1 indicating antagonism [30,31]. The software fitted the dose–response curves to determine the CI, and the conformity of the data to the fitted curves is indicated by the correlation coefficient (r). The r values obtained for each combination are presented in Table 1.

**Figure 2:**
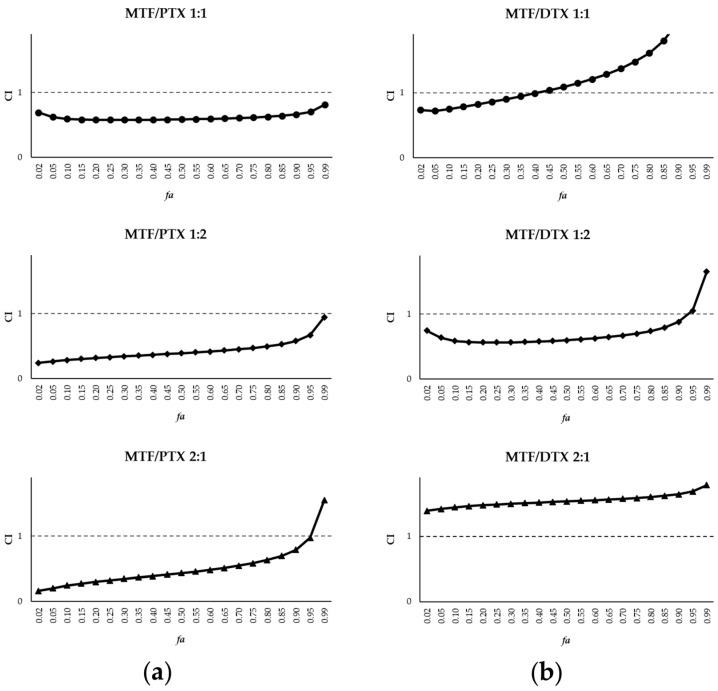
Interaction effect of MTF/PTX (**a**) and MTF/DTX (**b**) combinations (1:1, 1:2 and 2:1) on iRFP-*L. infantum* axenic amastigotes, expressed as CI versus *fa*, calculated using CalcuSyn software. Data represent the mean of three independent experiments, each with three technical replicates.

Figure 2 shows the CI values for MTF combined with PTX and DTX, plotted against the fraction of dead amastigotes (*fa*; fraction affected). For the MTF/PTX combinations, the 1:1 ratio was slightly synergistic, with CI values close to 1, whereas the 1:2 and 2:1 ratios showed lower CI values, remaining below 1 across the entire range of *fa* with the 1:2 combination showing the strongest effect (Figure 2a). In the case of DTX, the 1:1 combination exhibited synergism only at low *fa* values, while the 1:2 combination showed the most pronounced synergistic effect (Figure 2b).

CalcuSyn software also determines the dose reduction index (DRI), which quantifies the extent to which the dose of each drug in a synergistic combination can be reduced while maintaining a given level of effect [31]. A DRI value > 1 indicates a favourable dose reduction, whereas values close to or <1 suggest little or no advantage in dose reduction. The DRI values obtained for PTX and DTX in combination with MTF at different effect levels (fraction of dead amastigotes) are presented in Table 1. Overall, MTF/PTX combinations yielded higher DRI values than those observed for MTF/DTX combinations (Table 1).

**Figure 3:**
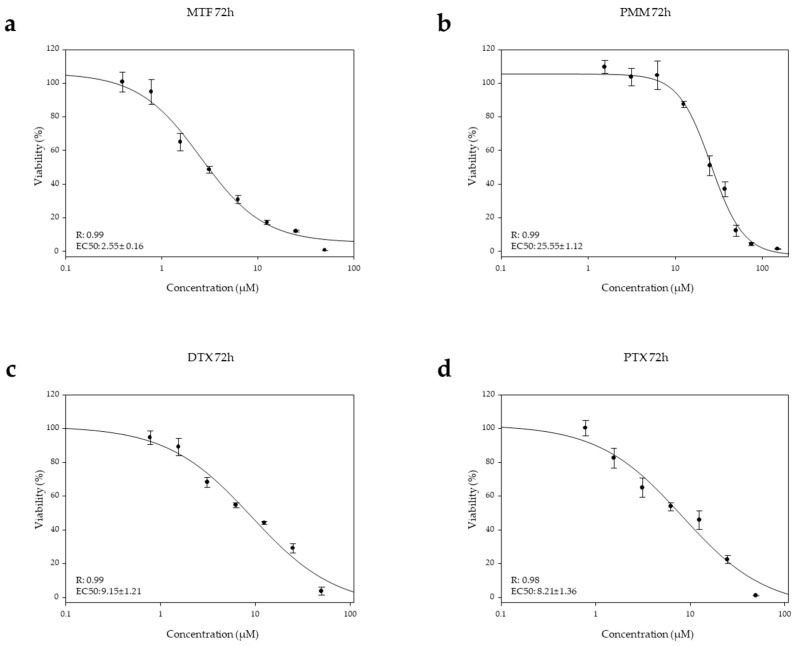
Dose–response curves of MTF (**a**), PMM (**b**), DTX (**c**) and PTX (**d**) against iRFP-*L. infantum* intramacrophagic amastigotes obtained from splenic explants of infected Balb/c mice. Serial ^1^/_2_ dilutions of MTF, DTX and PTX were prepared from a starting concentration of 50 µM, while PMM was tested using both ^1^/_2_ and ^2^/_3_ serial dilutions starting from 150 µM. Dose–response curves were fitted using SigmaPlot software. Data are presented as mean ± SD of three independent experiments with five technical replicates each.

The combination effect of taxanes with MTF was further evaluated using intramacrophagic amastigotes. Based on the results obtained in the previous experiments, the combination 1:2 was selected for assessment. The EC_50_ values obtained for DTX and PTX were 9.15 ± 1.21 µM and 8.21 ± 1.36 respectively (Figure 3c,d), whereas the EC_50_ value for MTF in this platform was 2.55 ± 1.01 µM (Figure 3a).

**Figure 4:**
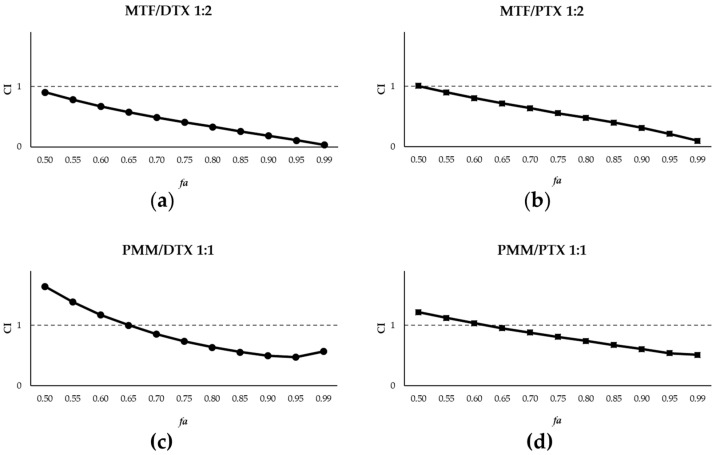
Interaction effects of MTF/taxane (1:2) combinations with DTX (**a**) and with PTX (**b**) and PMM/taxane (1:1) combinations with DTX (**c**) and with PTX (**d**) on iRFP-*L. infantum* intramacrophagic amastigotes, expressed as CI versus *fa*, calculated using CalcuSyn software. Data represent the mean of three different experiments, each with three technical replicates.

The CI values obtained for these combinations are represented in Figure 4a,b. Synergistic interactions were observed at *fa* values > 0.5. In this case, the DRI values for DTX at *fa* values above 75% were greater than those obtained for PTX (Table 2).

### 2.2. Leishmanicidal Effect of PTX and DTX in Combination with PMM

Given the limited effect of PMM against axenic amastigotes [15,16], the combination of taxanes with PMM was evaluated exclusively in intramacrophagic amastigotes. The EC_50_ value for PMM in this system was 25.54 ± 1.12 µM (Figure 3b). Based on the EC_50_ value obtained, a 1:1 combination ratio was selected for both taxanes. As shown in Figure 4c,d, synergistic interactions occurred at higher *fa* values for both PTX and DTX, with DRI values > 75%, *fa* being higher for DTX than for PTX (Table 2).

### 2.3. Cytokine Evaluation

To study whether taxanes promote an immune response favourable to parasite killing, we analysed the cytokines secreted by splenocytes derived from infected Balb/c mice. Splenocytes were treated with these drugs either alone or in combination, using the previously established ratios of 1:2 for MTF/taxane and 1:1 for PMM/taxane. After 72 h of treatment, and subsequent fluorescence measurement, culture supernatants were collected from the assay plates. Two concentrations were selected for cytokine analysis based on parasite mortality and splenocyte viability data. Cytokines were therefore determined at the following concentrations of the dose–response curves: 12.5 and 6.25 µM MTF alone; 25 and 12.5 µM PMM alone; 25 and 12.5 µM taxane alone; 12.5 + 25 µM MTF/taxane; 6.5 + 12.5 µM MTF/taxane, 25 + 25 µM PMM/taxane and 12.5 + 12.5 µM PMM/taxane.

**Figure 5:**
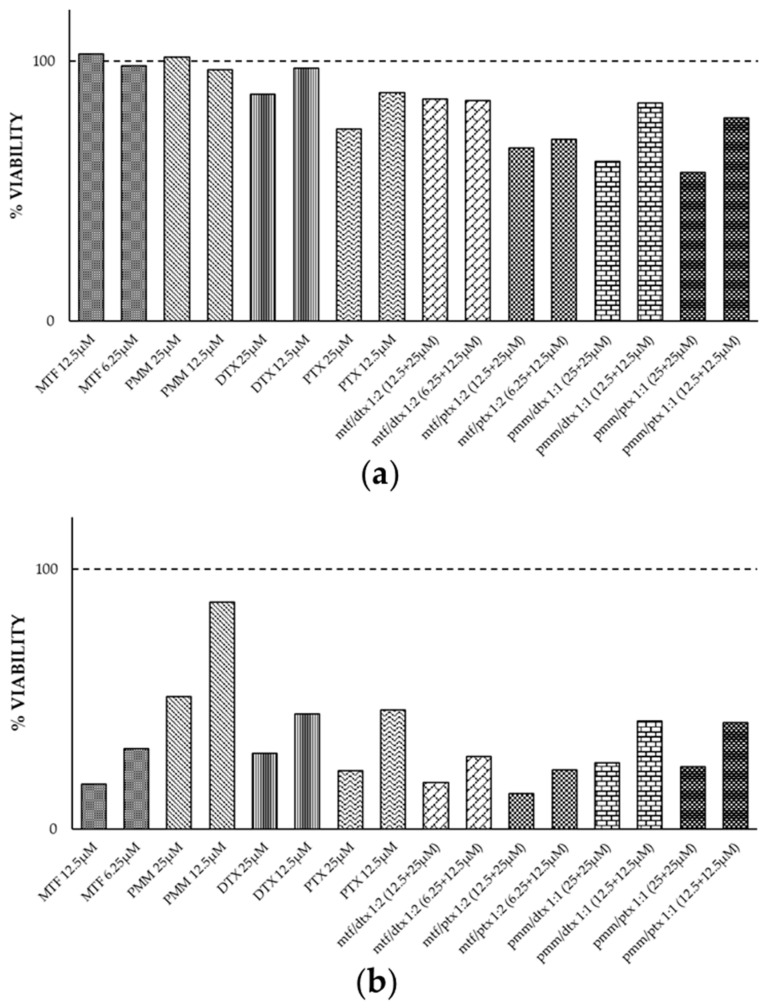
(**a**) Cell viability of primary splenic cultures from uninfected Balb/c mice exposed to MTF, PMM, DTX, PTX, and their combinations at the concentrations selected for cytokine evaluation. Viability was assessed using the Alamar Blue assay, with negative control wells (0.1% DMSO) set as 100%. Data represent the mean of three independent experiments each with three technical replicates. (**b**) Intramacrophagic amastigote viability after exposure to MTF, PMM, DTX, PTX and their combinations at the concentrations selected for cytokine evaluation. Data represent the mean of three different experiments, each with three technical replicates.

Cytotoxicity of the individual compounds and their combinations was assessed using primary splenic cultures obtained from uninfected Balb/c mice. These cultures were exposed to drugs alone or in combination, and cell viability was determined using the alamarBlue (Invitrogen) assay as described in the Section 4. Figure 5a shows viability values obtained for the selected concentrations, while Figure 5b shows parasite viability.

#### 2.3.1. Effect of PTX and DTX in Combination with MTF

**Figure 6:**
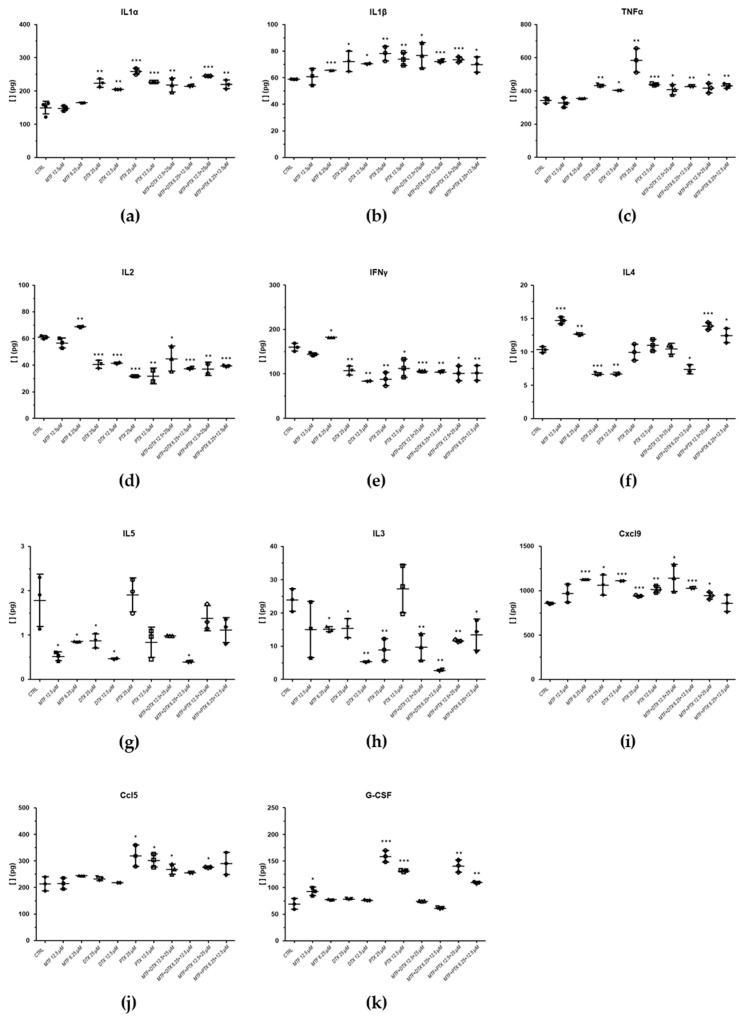
Cytokine values obtained from infected splenocyte cultures, exposed to MTF, DTX, PTX, MTF/DTX (1:2) combination, and MTF/PTX (1:2) combination, compared with the control (splenocytes isolated from infected mice and treated with 0.1% DMSO). (**a**) IL-1α; (**b**) IL-1β; (**c**) TNF-α; (**d**) IL-2; (**e**) IFN-γ; (**f**) IL-4; (**g**) IL-5; (**h**) IL-3; (**i**) Cxcl9; (**j**) Ccl5; (**k**) G-CSF. Statistical analysis was performed using Student’s *t*-test. Significance levels are indicated by asterisks (* < 0.05; ** < 0.01; *** < 0.001).

Figure 6 shows cytokine levels with significant differences in treated samples compared to the control (splenocytes infected and treated with 0.1% DMSO), as determined by Student’s *t*-test.

In samples treated with taxanes, we observed a significant increase in cytokines associated with parasite clearance, promoting macrophage polarisation toward the M1 phenotype such as IL-1α, IL-1β and TNF-α [32]. Treatments with both concentrations of PTX and DTX alone resulted in a significant increase in IL-1α compared with the control; the levels of this cytokine in the combinations were similar to those observed with taxanes alone, while MTF treatment did not induce changes in IL-1α (Figure 6a). A similar pattern was observed for IL-1β, with the additional observation that IL-1β levels were also significantly elevated in one of the samples exposed to MTF (Figure 6b). TNF-α, a key factor in disease control that enhances macrophage activity and parasite elimination, was significantly increased relative to the control in samples treated with taxanes. Combination treatments with MTF did not further enhance TNF-α levels compared with taxanes alone, and MTF treatment did not induce changes compared with the control (Figure 6c).

IL-2 plays a central role in stimulating IFN-γ production and activating T and NK cells [33,34], but its production has also been implicated in limiting Th1 response establishment via a regulatory mechanism independent of conventional Treg cells [35]. In our study, IL-2 levels were significantly reduced in samples treated with taxanes and their combinations compared with the control, whereas no significant changes were observed following MTF treatment (Figure 6d).

Regarding IFN-γ, treatment with taxanes and their combinations resulted in a significant decrease in its levels (Figure 6e). However, the chemokine Ccl5, which is upregulated by IFN-γ, was increased in three of the samples treated with PTX and in one sample treated with a DTX combination (Figure 6j). Similarly, Cxcl9, another chemokine regulated by IFN-γ, showed increased levels following treatment with 6.25 µM MTF and both concentrations of PTX and DTX compared with the control (Figure 6i). These results suggest that IFN-γ may have been upregulated at an earlier stage of the experiment. Both Ccl5 and Cxcl9 are associated with the recruitment of Th1 cells and protection against the parasite [36].

IL-4 and IL-5 are associated with suppression of Th1 cells and macrophage deactivation [33,34]. Regarding IL-4, DTX reduced its levels, whereas MTF treatment resulted in an increase at the tested concentrations (Figure 6f). No changes were observed in samples treated with PTX alone and combinations involving PTX exhibited a pattern similar to that of MTF (Figure 6f). Concerning IL-5 levels, DTX, MTF, and their combinations significantly reduced levels compared to the control (Figure 6g). The effect of PTX was less consistent, with no significant differences observed (Figure 6g).

IL-3 has been associated with both disease progression and parasite clearance. In the context of disease progression, macrophages differentiated under IL-3 stimulation have been reported to promote Th2 cells expansion [37]. Although high variability was observed in IL-3 levels in some samples (12.5 µM MTF; 12.5 µM PTX), other treatments resulted in significant decreases compared with the control (Figure 6h). PTX induced the expression of granulocyte colony-stimulating factor (G-CSF), which is an important regulator of neutrophil activity (Figure 6k) [38].

#### 2.3.2. Effect of PTX and DTX in Combination with PMM

**Figure 7:**
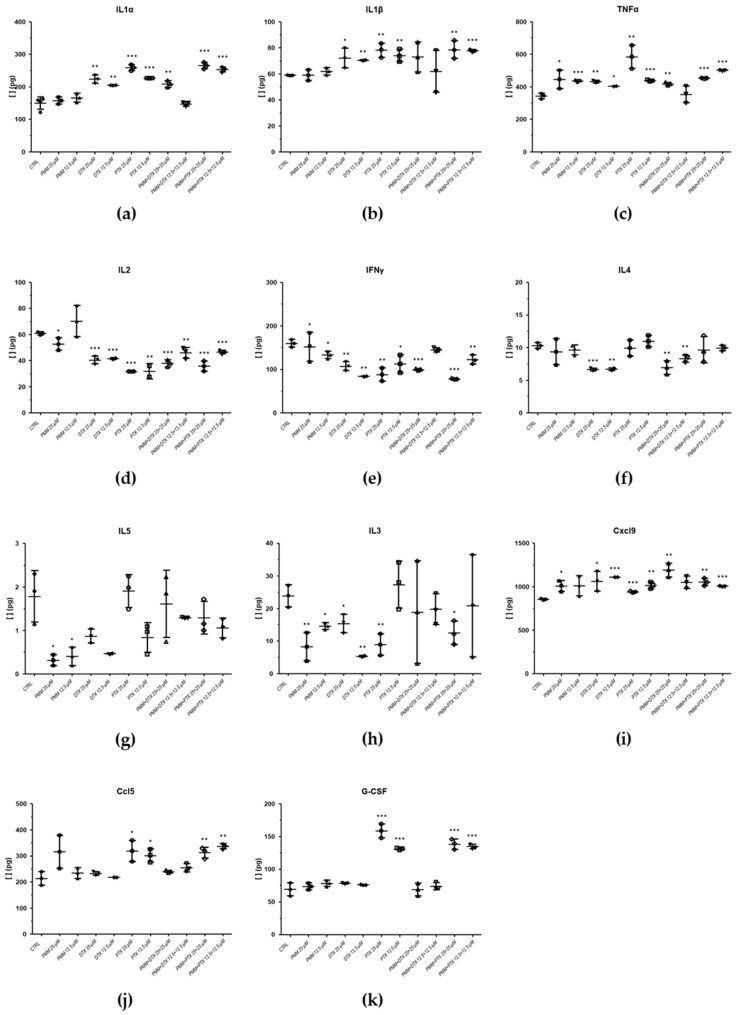
Cytokine values in infected splenocyte cultures, exposed to PMM, DTX, PTX, PMM/DTX (1:1), and PMM/PTX (1:1) combinations, compared with the control (splenocytes isolated from infected mice and treated with 0.1% DMSO). (**a**) IL-1α; (**b**) IL-1β; (**c**) TNF-α; (**d**) IL-2; (**e**) IFN-γ; (**f**) IL-4; (**g**) IL-5; (**h**) IL-3; (**i**) Cxcl9; (**j**) Ccl5; (**k**) G-CSF. Statistical analysis was performed using Student’s *t*-test. Significance levels are indicated by asterisks (* < 0.05; ** < 0.01; *** < 0.001).

Samples exposed to PMM and its combinations with taxanes exhibited similar patterns to those described for IL-1α, IL-1β, IL-2, IFN-γ cytokines in their combinations with MTF (Figure 7). For IL-1β, none of the samples treated with PMM/DTX combinations showed a significant increase in cytokine levels (Figure 7b). PMM and taxanes alone increased TNF-α levels compared with the control, whereas combination treatments did not further enhance TNF-α content (Figure 7c).

No changes in IL-4 levels were observed in samples treated with PMM or PMM/PTX combinations compared with the control (Figure 7f), while PMM/DTX combinations decreased IL-4 levels similarly to DTX alone (Figure 7f). PMM treatment also reduced IL-5 and IL-3 levels at the tested concentrations (Figure 7g,h). In contrast to PTX, DTX and PMM/DTX combinations did not affect G-CSF levels (Figure 7k).

## 3. Discussion

Drug combination and drug repurposing are two strategies increasingly employed in the search for novel therapies across a broad range of diseases. Combination therapies can reduce required drug dosages and help overcome drug resistance. Meanwhile, drug repurposing aims to identify new therapeutic applications for molecules already approved for other indications, thereby reducing both the time and costs associated with developing of new treatments. Furthermore, the dose reduction afforded by combination regimes may enhance the success of these repurposing strategies [15,16].

Taxanes, comprising the natural product paclitaxel (PTX) and its semi-synthetic analogue docetaxel (DTX), represent a class of potent antineoplastic agents that stabilise microtubules by inhibiting depolymerisation and disrupting cellular dynamics—a mechanism that also underpins their documented antileishmanial activity [17,18,19,39]. However, their therapeutic application remains constrained by systemic toxicity and the emergence of resistance as observed in oncological settings [40,41]. These limitations make taxanes compelling candidates for synergistic intervention studies. Consequently, we evaluated the effects of PTX and DTX in combination with MTF and PMM—two standard drugs used for the treatment of leishmaniasis—that are themselves associated with significant adverse side effects.

Our in vitro and ex vivo studies demonstrated synergistic interactions between taxanes and both miltefosine (MTF) and paromocycin (PMM). Dose reduction index (DRI) quantifies the magnitude of dose reduction achievable for each drug in combination compared to its monotherapy equivalent (at a specific *fa*). In the ex vivo studies, at *fa* values exceeding 75%, we observed DRI values > 1 for MTF, PMM and taxanes, indicating favourable dose reductions; these were particularly pronounced with DTX (DRI > 71). Therefore, these combinations allow for a reduction in the required dosages of MTF, PMM and taxanes, to obtain the antiparasitic effect, thereby reducing the toxicity associated with monotherapies.

Beyond direct antiparasitic activity, another critical research area in the development of safer and more effective antileishmanial treatments focuses on the induction of a favourable host immune response. Taxanes and in particular PTX have been reported to activate macrophages into a pro-inflammatory phenotype by binding to toll-like receptor 4 (TLR4) on the macrophage cell surface [42]. In this regard, previous studies have shown that exposure of *L. major*-infected murine macrophages to PTX enhances parasite clearance through macrophage activation mediated by NO [27].

Different aspects of both innate and adaptive immune responses are compromised during *Leishmania* infection. Among them, the so-called ‘Th1–Th2 dichotomy’ is particularly relevant, as the balance between the Th1 and Th2 responses largely determines the clinical course of the infection. Resistance to the parasite has been associated with the activation of the Th1 response, mediated by IL-12, which promotes the production of IFN-γ and TNF-α by CD4+ and CD8+ T lymphocytes, favouring the polarisation of M0 macrophages towards the M1 phenotype. M1 macrophages release reactive oxygen species and NO, leading to parasite killing. In contrast, activation of the Th2 response—characterised by the secretion of IL-4, IL-10, IL-13, or TGF-β cytokines and by macrophage polarisation toward the M2 phenotype—has been associated with disease progression [43,44,45,46,47]. Although this dichotomy has been extensively described, accumulating evidence indicates that immune regulation is more complex than initially proposed, with the roles of certain cytokines varying depending on the *Leishmania* species, the host strain, and the affected organs [34].

In this context, macrophages and their polarisation into distinct functional phenotypes play a pivotal role in the immune response. The expression of TNF-α and IFN-γ by macrophages, Th1 cells, and CD8+ T lymphocytes can lead to the polarisation of M0 macrophages toward the M1 phenotype, which is characterised by the secretion of TNF-α, IL-1α, IL-1β, IL-6, IL-12, and Cxcl9 [32]. Our results suggest that PTX and DTX exert immunomodulatory effects that favour Th1 responses and M1 macrophage polarisation, as evidenced by significantly increased TNF-α, IL-1α, and IL-1β levels in taxane-treated samples compared to controls. Although IFN-γ levels were not significantly elevated at the analysed time point, we observed increased concentrations of IFN-γ-regulated chemokines, such as Cxcl9 and Ccl5 [36,48]. This suggests that IFN-γ upregulation may have occurred at earlier stage. Both taxanes increased Cxcl9 levels, a chemokine associated with Th1 cell recruitment and the reduction of parasite load [49]. In addition, Ccl5—which was elevated in PTX-treated samples—is typically produced at high levels during Th1 responses [36] and has been implicated in the control of experimental infections with *L. major* [50] and *L. braziliensis* [51]. Consistent with these findings, the chemotactic role of Ccl5 has been well-documented in VL, where it facilitates the recruitment of mononuclear cells and mediates effective hepatic immune control in C57BL/6 mice [48]. However, its involvement in the resolution of canine VL remains controversial [52].

Our results also showed that taxanes led to a reduction in IL-2 levels. Previous studies using experimental infection models have indicated that *L. donovani* decreases IL-2 expression, which was initially interpreted as a mechanism contributing to disease progression due to the role of IL-2 in inducing IFN-γ production and T-cell activation [53]. However, it has been also described that during the early phase of experimental VL, IL-2 can induce both IFN-γ and IL-10. The induction of IL-10 may subsequently suppress IL-2 production, leading to the establishment of infectious tolerance [53]. The limitation of IL-2-mediated Th1 responses has also been attributed to regulatory mechanisms that function independently of Treg cells [35]. Consequently, the observed taxane-induced reduction in IL-2 might counteract this early suppression, thereby preventing the premature inhibition of the Th1 response.

The decrease in IL-4 and IL-5 levels, more clearly observed in DTX-treated samples, is consistent with the proposed role of taxanes in promoting Th1 activation and M1 macrophage polarisation. IL-4 has been associated with disease progression in experimental infections through the induction of humoral immune responses, macrophage deactivation and suppression of Th1 cells [34,54]. Elevated levels of IL-4 and IL-5 have also been reported in samples from patients during the acute phase of VL compared to healthy controls [55].

The role of IL-3 in leishmaniasis remains controversial, as it has been linked to both disease progression and parasite clearance [33,44]. In the present study, reduced IL-3 levels were observed in samples exposed to MTF, PMM, PTX, DTX, and their combinations compared to controls, suggesting that IL-3 expression might be associated with infection progression. Furthermore, previous studies have shown that macrophages differentiated under IL-3 stimulation favour the expansion of Th2 cells [37].

In response to pro-inflammatory cytokines, monocytes, T cells, and macrophages produce G-CSF, which enhances neutrophil responses. However, G-CSF signalling must be tightly regulated to prevent excessive neutrophil activation and subsequent tissue damage [38]. Consequently, systemic G-CSF administration increases serum levels of IFNα and IL-10, while inhibiting IL-1β, IL-12, IFN-γ, and TNF-α [56]. Therefore, G-CSF has been associated with epithelialisation and healing processes in CL, and evaluated in combination therapy in experimental models of the disease [57]. On the other hand, in experimental models of VL, G-CSF-driven neutrophilia has been linked to immune regulation and parasite persistence rather than effective parasite clearance [58]. PTX has been reported to induce G-CSF expression via NF-κB-dependent pathways [59], which may explain the increased G-CSF levels observed in PTX-treated samples—reflecting an inflammatory environment that had not yet downregulated at the time of the analysis.

Taken together, our data indicate that PTX and DTX exert a favourable immunomodulatory effect by promoting Th1 response activation and M1 macrophage polarisation. The fact that the addition of MTF or PMM did not further enhance this specific modulation beyond the levels achieved by taxane monotherapy, suggests a ‘ceiling effect,’ likely due to the maximal activation of pro-inflammatory pathways by taxanes alone. Consequently, future research utilising murine experimental models is essential to evaluate the therapeutic efficacy and clinical potential of these taxane-based combinatorial regimens.

## 4. Materials and Methods

### 4.1. Drugs

Docetaxel (DTX) and paclitaxel (PTX) (Thermo Fisher Scientific, Waltham, MA, USA) were dissolved in dimethyl sulfoxide (DMSO; Thermo Fisher Scientific, USA) to obtain stock solutions at the final concentration of 100 mM. Miltefosine (MTF) and paromomycin (PMM) (Thermo Fisher Scientific, Waltham, MA, USA) were dissolved in sterile H_2_O to prepare stock solutions at concentrations of 50 mM and 100 mM, respectively.

### 4.2. Experimental Animals and Ethical Statement

Six-week-old female Balb/c mice were obtained from Janvier Laboratories (St Berthevin Cedex, France) and housed in the Animal Facility of the University of León (Spain) under standard housing conditions with free access to food and water. All animal procedures were conducted in accordance with Spanish Legislation (RD 118/2021, which modifies RD 53/2013) in compliance with European Union Directive (EU 2019/1010). The experimental protocols were reviewed and approved by the Junta de Castilla y León (approval number OEBA-ULE-004-2025; approval date: 9 April 2025).

### 4.3. Parasites

The iRFP-*L. infantum* strain was used for in vitro and ex vivo studies. This strain derives from *L. infantum* BCN150 (MCAN/ES/96/BCN 150) and was genetically engineered in-house to constitutively produce the infrared fluorescent protein (iRFP). This modification enables near-infrared detection of viable parasites [60,61]. Promastigotes were grown in Schneider’s insect medium (Sigma-Aldrich, Merck, Darmstadt, Germany) supplemented with 20% fetal calf serum (FBS) and antibiotic cocktail (100 U/mL penicillin and 100 µg/mL streptomycin) at 26 °C under continuous agitation. iRFP-*L. infantum* metacyclic promastigotes were used for mouse infection.

### 4.4. Experimental Infections and Set up of Primary Cultures

Eight-week-old female Balb/c mice were inoculated intraperitoneally with 2.5 × 10^9^ metacyclic promastigotes of iRFP-*L. infantum*. Between 8 to 12 weeks after inoculation, mice were humanely sacrificed to aseptically obtain infected spleens, as well as the femur and tibia of both hind limbs.

Femur and tibia were used for the isolation of axenic iRFP-*L. infantum* amastigotes. Briefly, both ends of each bone were cut and bone marrow cells were flushed from the medullary cavity using a 27-gauge needle filled with pre-warmed (37 °C) 1× PBS. The resulting cell suspension was passed through a 100 µm cell strainer and centrifuged at 3500 rpm for 10 min at room temperature. The pellet was resuspended in and incubated at 37 °C and 5% CO_2_ to allow differentiation into axenic amastigotes. The composition of the amastigote culture medium was: 15 mM KCl; 136 mM KH_2_PO_4_; 10 mM K_2_HPO_4_·3H_2_O; 0.5 mM MgSO_4_·7H_2_O; 24 mM NaHCO_3_; 22 mM glucose; 1 mM glutamine; 1× RPMI 1640 vitamin mixture (Sigma-Aldrich, Merck, Darmstadt, Germany); 10 mM folic acid; 100 mM adenosine; 1× RPMI amino acid mixture (Sigma-Aldrich, Merck, Darmstadt, Germany); 5 mg/mL hemin; antibiotic cocktail; 25 mM MES; and 20% FBS.

Infected spleens were minced into small fragments and incubated for 25 min with 5 mL of 2 mg/mL collagenase D (Merck, Darmstadt, Germany) dissolved in buffer (10 mM HEPES, pH 7.4, 150 mM NaCl, 5 mM KCl, 1 mM MgCl_2_, and 1.8 mM CaCl_2_). The resulting suspension was mechanically dissociated by passage through a 100 µm cell using pre-warmed (37 °C) 1× PBS. Cells were centrifuged at 1800 rpm for 7 min at 4 °C and incubated in ice for 4 min in erythrocyte lysis buffer (150 mM NH_4_Cl; KHCO_3_; EDTA 0.1 mM). The lysis reaction was stopped by adding pre-warmed (37 °C) and cells were washed three times by centrifugation at 1800 rpm for 7 min at 4 °C, washing with 1× PBS between each centrifugation.

The final cell suspension containing infected macrophages, was resuspended in RPMI medium (Gibco, Thermo Fisher Scientific, Waltham, MA, USA) supplemented with 20% FBS, 1 mM sodium pyruvate, 24 mM NaHCO_3_, 2 mM L-glutamine, 1× RPMI vitamins, 25 mM HEPES, and the antibiotic cocktail.

### 4.5. Axenic and Intramacrophagic Amastigotes Viability Assays

The antileishmanial effect of the compounds, both individually and in combination, was evaluated using black 384-well microtitre plates with optical bottoms. For axenic amastigote assays, 3 × 10^4^ iRFP-*L. infantum* parasites were seeded per well in 40 μL of amastigotes culture medium. A total of 40 μL of serial 1/2 or 2/3 dilutions of each compound or combinations, prepared in the same medium, was added to each well. Plates were incubated for 72 h at 37 °C under 5% CO_2_. Wells containing 0.1% DMSO and 10 μM AMB were used as negative and positive controls, respectively.

For intramacrophagic amastigote assays, 40 μL of murine splenocyte suspensions, obtained from infected mice as described above, were mixed with 40 μL of 1/2 or 2/3 dilutions of each drug, either alone or in combination, prepared in supplemented RPMI medium. Plates were also incubated at 37 °C under 5% CO_2_ for a period of 72 h. As for axenic amastigote assays, negative (0.1% DMSO) and positive controls (10 μM AMB) were included in all the experiments.

Cell viability of both axenic and intramacrophagic amastigotes was assessed by quantifying the fluorescence emitted at 700 nm by the iRFP protein, which is exclusively produced by living parasites, using the LiCor Odyssey^®^ 9120 infrared imaging system (LI-COR Biotech, LLC, Lincoln, NE, USA). Fluorescence values obtained from negative control wells (0.1% DMSO), were considered as 100% parasite viability.

EC_50_ values for each drug or combination were calculated by plotting the fluorescence emitted by viable parasites against drug concentration, using the non-linear fitting analysis provided by the Sigma Plot 10.1 statistical package.

### 4.6. Cell Cytotoxicity

Cytotoxicity assays were performed using splenic cell explants obtained from uninfected mice and prepared as previously described. Cells were counted microscopically and seeded in 96-well plates at a density of 1 × 10^6^ cells per well. Serial dilutions of the compounds and their combinations, both dissolved in supplemented RPMI medium, were added. Solutions of 0.1% H_2_O_2_ and 0.1% DMSO were used as positive and negative controls respectively. Plates were incubated for 72 h at 37 °C under 5% CO_2_. Cell viability was then assessed using the Alamar Blue assay (Invitrogen, Thermo Fisher Scientific, USA) following the manufacturer’s instructions. Absorbance was measured using a Varioskan™ LUX Multimode Microplate Reader (Thermo Fisher Scientific, USA). Cell viability was estimated by defining the absorbance values of the negative control wells as 100% viability. Data were evaluated using the non-linear regression analysis with the Sigma Plot 10.1 statistical package.

### 4.7. Cytokine Analysis

Supernatants from infected spleen explant cultures were collected 72 h after compound exposure for cytokine profiling, which was performed by COBIOMIC (Córdoba, Spain) using Olink^®^ Proximity Extension Assay (PEA) technology (OLINK Bioscience, Uppsala, Sweden); the cytokines included in the Target 48 Mouse Cytokine panel are listed online: https://cobiomicbioscience.com/wp-content/uploads/2025/07/Target-48-Mouse-Cytokine.pdf (accessed on 24 March 2026).

### 4.8. Statistical Analysis

The type of interaction obtained for the different drug combinations against axenic and intramacrophagic iRFP-*L. infantum* amastigotes was analysed using CalcuSyn software version 2.1 (Biosoft, Cambridge, UK, 1996–2007). Drug interactions were characterised using the combination index (CI)-median-effect isobologram equation. Isobologram analysis requires the construction of dose–response curves for each drug alone and in combination at multiple concentrations. These curves are defined by the parameters Dm (median-effect dose producing 50% inhibition), m (coefficient signifying the shape of the dose–response curve) and r (correlation coefficient indicating the conformity of the data with the curve). The statistical analysis for cytokines was performed using Student’s *t*-test. Levels of statistical significance were: * *p* < 0.05; ** *p* < 0.01; *** *p* < 0.001.

## Figures and Tables

**Table 1:** Correlation coefficient (r) and predictive dose reduction index (DRI) for MTF/taxane combinations tested in axenic amastigotes, calculated using the CalcuSyn. DRI indicates the fold reduction in the dose of each drug at a given effect level in synergistic combinations.

		DRI Values at Following Effect Levels							
		25%		50%		75%		95%	
Drug	r	MTF	Taxane	MTF	Taxane	MTF	Taxane	MTF	Taxane
**MTF + DTX 1:1**	0.84	1.74	3.48	1.16	4.44	0.77	5.66	0.39	8.50
**MTF + DTX 1:2**	0.98	3.54	3.55	2.54	4.87	1.82	6.67	1.04	11.33
**MTF + DTX 2:1**	0.74	0.91	3.64	0.75	5.73	0.61	9.02	0.44	19.35
**MTF + PTX 1:1**	0.91	2.29	7.04	2.04	10.20	1.82	14.79	1.50	27.59
**MTF + PTX 1:2**	0.92	4.14	11.10	3.22	12.33	2.50	13.69	1.64	16.33
**MTF + PTX 2:1**	0.92	3.82	23.47	1.92	19.16	0.96	15.64	0.30	11.12

**Table 2:** Correlation coefficient (r) and predictive dose reduction index (DRI) for MTF/taxane and PMM/taxane combinations tested in amastigotes obtained from splenic explants of infected Balb/c mice, calculated using CalcuSyn software. DRI indicates the fold reduction in the dose of each drug at a given effect level in synergistic combinations.

		DRI Values at Following Effect Levels							
		25%		50%		75%		95%	
Drug	r	MTF	Taxane	MTF	Taxane	MTF	Taxane	MTF	Taxane
**MTF/DTX 1:2**	0.999	0.58	1.24	1.46	4.54	3.04	12.67	10.41	71.17
**MTF/PTX 1:2**	0.97	0.53	3.34	1.23	5.19	2.38	7.35	7.23	13.20
**PMM/DTX 1:1**	0.99	5.30	0.16	4.13	0.71	3.38	2.27	2.42	15.87
**PMM/PTX 1:1**	0.96	5.01	0.55	4.32	1.01	3.73	1.85	2.91	5.10

## Data Availability

The raw data generated in this study have been deposited in Zenodo repository. DOI:10.5281/zenodo.18324944.

## Abbreviations

The following abbreviations are used in this manuscript:

AMB	amphotericin B
CI	combination index
CL	cutaneous leishmaniasis
Dm	median-effect dose producing 50% inhibition
DRI	dose reduction index
DTX	docetaxel
*fa*	fraction affected
L-AMB	liposomal encapsulated amphotericin B
m	coefficient signifying the shape of the dose–response curve
MCL	mucocutaneous leishmaniasis
MTF	miltefosine
PKDL	post-kala-azar dermal leishmaniasis
PMM	paromomycin
PTX	paclitaxel
r	correlation coefficient
VL	visceral leishmaniasis
